# In Vitro Tests of FDM 3D-Printed Diclofenac Sodium-Containing Implants

**DOI:** 10.3390/molecules25245889

**Published:** 2020-12-13

**Authors:** Petra Arany, Ildikó Papp, Marianna Zichar, Máté Csontos, János Elek, Géza Regdon, István Budai, Mónika Béres, Rudolf Gesztelyi, Pálma Fehér, Zoltán Ujhelyi, Gábor Vasvári, Ádám Haimhoffer, Ferenc Fenyvesi, Judit Váradi, Vecsernyés Miklós, Ildikó Bácskay

**Affiliations:** 1 Department of Pharmaceutical Technology, Faculty of Pharmacy, University of Debrecen, Nagyerdei Körút 98, H-4032 Debrecen, Hungary; arany.petra@pharm.unideb.hu (P.A.); feher.palma@pharm.unideb.hu (P.F.); ujhelyi.zoltan@pharm.unideb.hu (Z.U.); vasvari.gabor@pharm.unideb.hu (G.V.); haimhoffer.adam@pharm.unideb.hu (Á.H.); fenyvesi.ferenc@pharm.unideb.hu (F.F.); varadi.judit@pharm.unideb.hu (J.V.); vecsernyes.miklos@pharm.unideb.hu (V.M.); 2 Doctoral School of Pharmaceutical Sciences, University of Debrecen, Nagyerdei St. 98, H-4032 Debrecen, Hungary; 3 Department of Computer Graphics and Image Processing, Faculty of Informatics, University of Debrecen, Kassai út 26, H-4028 Debrecen, Hungary; papp.ildiko@inf.unideb.hu (I.P.); zichar.marianna@inf.unideb.hu (M.Z.); 4 Department of Physical Chemistry, Faculty of Science and Technology, University of Debrecen, Egyetem tér 1, H-4032 Debrecen, Hungary; csontos.mate@science.unideb.hu; 5 Science Port Kft., Varró utca 21, H-5300 Karcag, Hungary; elek@scienceport.hu; 6 Institute of Pharmaceutical Technology and Regulatory Affairs, University of Szeged, Eötvös u. 6, H-6720 Szeged, Hungary; geza.regdon@pharm.u-szeged.hu; 7 Faculty of Engineering, University of Debrecen, Ótemető utca 2-4, H-4028 Debrecen, Hungary; budai.istvan@eng.unideb.hu; 8 Department of Medical Imaging, University of Debrecen, Nagyerdei Krt. 98, H-4032 Debrecen, Hungary; beres.monika@med.unideb.hu; 9 Department of Pharmacology and Pharmacotherapy, University of Debrecen, Nagyerdei Körút 98, H-4032 Debrecen, Hungary; gesztelyi.rudolf@pharm.unideb.hu

**Keywords:** personalized medicine, implants, 3D printing, FDM, dissolution tests, cytotoxicity

## Abstract

One of the most promising emerging innovations in personalized medication is based on 3D printing technology. For use as authorized medications, 3D-printed products require different in vitro tests, including dissolution and biocompatibility investigations. Our objective was to manufacture implantable drug delivery systems using fused deposition modeling, and in vitro tests were performed for the assessment of these products. Polylactic acid, antibacterial polylactic acid, polyethylene terephthalate glycol, and poly(methyl methacrylate) filaments were selected, and samples with 16, 19, or 22 mm diameters and 0%, 5%, 10%, or 15% infill percentages were produced. The dissolution test was performed by a USP dissolution apparatus 1. A 3-(4,5-dimethylthiazol-2-yl)-2,5-diphenyl-2*H*-tetrazolium bromide dye (MTT)-based prolonged cytotoxicity test was performed on Caco-2 cells to certify the cytocompatibility properties. The implantable drug delivery systems were characterized by thermogravimetric and heatflow assay, contact angle measurement, scanning electron microscopy, microcomputed tomography, and Raman spectroscopy. Based on our results, it can be stated that the samples are considered nontoxic. The dissolution profiles are influenced by the material properties of the polymers, the diameter, and the infill percentage. Our results confirm the potential of fused deposition modeling (FDM) 3D printing for the manufacturing of different implantable drug delivery systems in personalized medicine and may be applied during surgical interventions.

## 1. Introduction

Personalized medicine may be used to provide patient-specific treatment during surgeries. Surgical implants and local drug delivery can be united, and their synergy incorporates surgical and medical treatment into a one-step medication that offers unique therapeutic advantages: the ability to raise the effectiveness, decrease the amount of the used active pharmaceutical ingredients (APIs), minimize adverse effects, and increase patient compliance [1,2]. In orthopedic surgeries, mostly antibiotics, e.g., gentamicin-containing poly(methyl methacrylate) (PMMA) beds, pearls, or sponges, are used to locally release the API into the surrounding tissues [3]. Based on an experiment, customized implants in orthopedic surgeries can reduce the treatment time and improve the medical outcomes [4].

The application of 3D printing technology may be an alternative way to design effective, customized API combinations for the patient immediately [5]. Complex, personalized products can be prepared on-demand through a low-cost manufacturing process by healthcare providers [6]. Nevertheless, some limitations should be taken into consideration. The APIs used have to be stable during printing, which means they must have high heat tolerance, stability in different solvents, compatibility with cross-linking polymers, and tolerance to UV light. Although it is a fast prototyping technique, it is not able to exceed the productivity of an industrial-sized tableting machine [7].

One of the most studied 3D printing techniques is fused deposition modeling (FDM) or fused filament fabrication (FFF) [8], which is a process that places a molten polymer layer-by-layer on top of an already printed layer to build a desired object [9]. FDM technology is an off-patent, widespread, and inexpensive technology that has inspired many researchers to study the benefits of this technique in a modern healthcare system and in the pharmaceutical sciences to manufacture drug delivery systems [10,11]. Implantable drug delivery systems can be formulated in many different ways with 3D technologies. A research team manufactured a 3D-printed biodegradable implant for the personalized, local treatment of osteosarcoma [12]. In another study, implantable nanogels were manufactured to provide a long-lasting effect [13]. Some authors have found that 3D printing can provide an adequate scaffold for implantable bone purposes because the polymers can be combined with different APIs [14].

In the FDM printing, there are many parameters that influence the quality of the product—for example, the properties of the materials, the chamber, the extruder, and the deposition [15]. It is essential to examine the influence of the process parameters [16] and to determine the critical process parameters during the manufacturing of a drug delivery system [17]. For example, two different research groups examined the effect of the wall thickness on the dissolution profile in the case of 3D-printed capsules and shell–core delayed-release tablets. They found that shell thickness, as a printing parameter, plays an important role in the dissolution profile [18,19].

In most cases, researchers incorporate the API into the filament with hot-melt extrusion, and then, the API-containing filament is printed with FDM technology. In a review, the same approach was used: the API was hot-melt extruded and then the filament was 3D-printed [20]. The high extrusion temperature used in FDM (more than 120 °C) and higher temperature (above 150 °C) used to hot-melt extrude the filament mean that only heat-stable APIs can be used for direct printing [2]. Modified povidone granulated polymer was used to provide low-temperature FDM printing. In this case, the polymer granules have to be hot-melt extruded first with the model API of ramipril, whose melting point is around 109 °C [21]. It was also investigated how different added excipients can decrease the printing temperature, but the lowest adequate printing temperature was 165 °C [22]. Even though many research projects are trying to solve the problem of high printing temperature, the most promising solution would be to manufacture a carrier system in which all types of API can be incorporated.

In our study, polylactic acid (PLA), antibacterial PLA, polyethylene terephthalate glycol (PETG), and poly(methyl methacrylate) (PMMA) were used for the 3D printing. Polylactic acid (PLA) is a well-known and widely used polymer due to its thermoplasticity, biocompatibility, biodegradability, and useful mechanical properties [23], but the rigidity and brittle properties of the polymer must also be considered [24]. One type of PLA filament modification is antibacterial PLA, which is a commercially available filament that has an antibacterial effect due to the dispersed silver nanoparticles [25].

Another widely used filament is polyethylene terephthalate glycol (PETG). This polymer has high tensile strength and excellent resistance to chemicals. It is a nonbiodegradable, cheap, and glass-like transparent polymer [26]. Even though PETG is more susceptible to UV light damage and cannot be autoclaved, these drawbacks are relatively minor in comparison with the benefits [27].

Poly(methyl methacrylate) (PMMA) is a commercially available, relatively cheap, thermoplastic, nonbiodegradable, nontoxic, and inert polymer [28]. PMMA can be considered as a tissue material equivalent to the human body [29].

Thermogravimetric analysis (TGA) was performed to determine the thermal stability of the polymeric samples between 25 and 500 °C. Heatflow (Differential Scanning Analyis, DSC) analysis was performed to find the endothermal or exothermal difference [30]. These methods were used to gain information about the API or excipients affecting the polymer filament [22,31,32]. Contact angle measurements can provide information on the hydrophilicity and hydrophobicity of the surface, which is influenced by the polymer type and the polymer modifications [33].

The material structure of the printed samples can be examined by scanning electron microscopy (SEM) before and after the dissolution test in order to study the role of polymer porosity and the influence of pore size on the dissolution [34]. For example, a research group examined chitin‒PLA-laminated implantable film by SEM to determine the surface properties [23], and another research group investigated scaffolds composed of polylactide-co-glycolide (PLGA) and hydroxyapatite (HA) [35]. Microcomputed tomography (MicroCT) is an applicable method to detect and quantify porosity or defects and to visualize it in 3D images [36]. 3D-printed cartridges filled with pastes were examined to determine whether air bubbles are incorporated to the sample and gave information about the whole geometry and design [17]. The content uniformity of the printed systems was determined by Raman spectroscopy to specify the location and distribution of diclofenac sodium in the printed samples with different infill percentages [37]. 3D-printed discs were printed and filled with nitrofurantoin and the solid-state form of the API was determined by Raman spectroscopy. This technique is a potential process analytical tool for safeguarding the robust production of different 3D-printed geometries [38].

The biocompatibility properties of the printed samples were measured by a prolonged 3-(4,5-dimethylthiazol-2-yl)-2,5-diphenyl-2*H*-tetrazolium bromide dye (MTT) cytotoxicity test. MTT assay is a broadly used, rapid colorimetric method to measure the in vitro cytotoxicity of certain compounds on cell lines or primary cells [39]. Several assays may be used, such as MTT assay, LDH test, real-time cell electronic sensing assay (RT-CES), etc. [40], but the ISO standard determines the MTT test and its parameters, such as the number of cells in the dishes, the exposure period, and the type of positive control [41].

In the dissolution test, PLA-, antibacterial PLA-, PETG-, and PMMA-based samples with a diameter of 16, 19, or 22 mm were examined at 0%, 5%, 10%, or 15% infill rates to determine how these parameters influence the dissolution profile of diclofenac sodium as a model drug [42]. This API can be easily measured with UV-VIS spectrophotometry due to its good water solubility at a neutral pH [43]. Diclofenac sodium is a Biopharmaceutics Classification System (BSC) type II API, and many research groups use it as a model drug for the determination of the dissolution profile. Diclofenac sodium is an anti-inflammatory agent that provides local or systematic effects depending on the usage site, e.g., suppository, tablet, capsule, or injection [44]. Diclofenac sodium can also be used in an implantable drug delivery system to locally decrease the inflammatory response and prevent rejection [45].

Overall, the objective of the study was to design and test 3D-printed implantable drug delivery systems, which enables the use of any kind of API depending on the patient’s need. This article focuses on the optimization of printing parameters, i.e., avoiding the loss and decomposition of API through the printing process, and the drug delivery system can be printed at the bedside or in the operating room. Our samples were manufactured from different biodegradable and nonbiodegradable polymers to describe their structures and to investigate how the sample size and infill percentage (as printing parameters) may influence the dissolution profile and kinetics of the model drug.

## 2. Results

### 2.1. Design of the Drug Reservoirs and Printing of the Samples

In our experiments, four different polymers, polylactic acid (PLA), antibacterial PLA (Anti), polyethylene terephthalate glycol (PETG), and poly(methyl methacrylate) (PMMA), were used for the 3D printing. The sample thickness was 2.4 mm and the diameter was 16, 19, or 22 mm. The infill percentage rates were 0%, 5%, 10%, and 15%.

**Figure 1:**
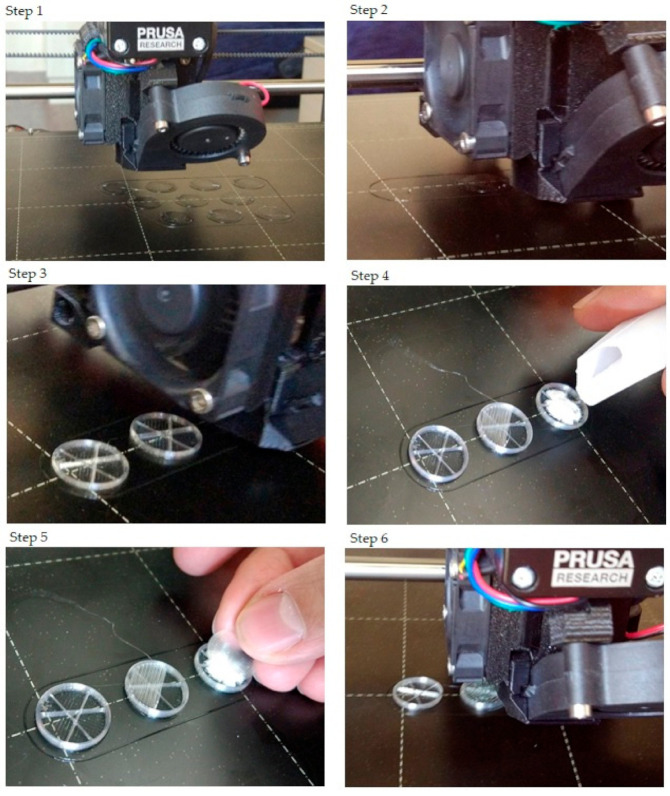
The step-by-step printing process. For the illustration, just three samples were printed, and only one sample was filled with diclofenac sodium salt. Step 1: In preparation, the extra covers should be printed. Their design was a cylinder with a determined diameter. The height was 0.2 mm, which was exactly one layer according to our settings. Step 2: The start of the printing and the first few layers. Step 3: After some layers, the structure of the infill pattern was already visible. The layer order was: two bottom layers (contour and full infill), five layers with contour and infill rate of 5% (in this case), and three layers without infill. Step 4: After printing the three layers without infill, the printing process should be paused. The printing head moved into the starting position, so the diclofenac sodium could be poured. Step 5: The powder was covered with the preprinted covering part. Step 6: The final step was to print two top layers that close the sample.

**Figure 2:**
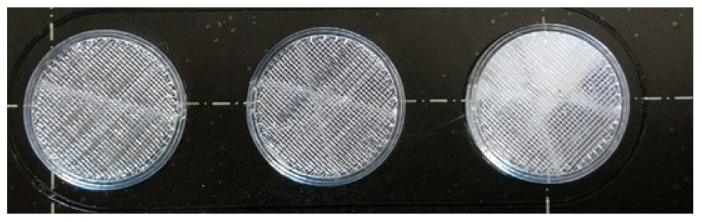
Image of the printed samples. In this case, only the sample in the right-hand corner contains diclofenac sodium.

The step-by-step printing process is presented in Figure 1. First, the filaments were melted to the desired temperature. In the case of PLA and antibacterial PLA, the printing temperature was 215 °C; PETG was printed at 250 °C, and PMMA was printed at 270 °C. Our research group developed an easy method to fill the samples without the loss of API, so an extra covering part is printed separately (Figure 1, step 1). The sample printing took place, and at the appropriate moment, the process was stopped (Figure 1, steps 2 and 3); then, the API was poured (Figure 1, step 4), and the preprinted extra covering part was placed in the top of the API (Figure 1, step 5). Finally, the top two layers were printed (Figure 1, step 6). In this way, neither the extrusion temperature nor the ventilation disturbed the FDM process. The printed structures are presented in Figure 2.

The samples were labeled with the polymer used (e.g., PLA), the diameter of the sample (e.g., 16), and finally, the sample’s infill percentage (e.g., 0); in this case, the sample name is PLA_16_0.

### 2.2. Content Uniformity and Weight Variation

The measured weight and the content uniformity results can be seen in Table 1. The samples’ average weight (mg) depended on the polymer and increased with the increase in diameter and infill percentage. The samples were filled with ~30 mg of diclofenac sodium salt.

### 2.3. PLA Degradation

As PLA and antibacterial PLA are biodegradable polymers, the degradation amount of the samples was determined at pH = 7.4, 37 °C. The degradation was determined by weight measurement, but during the examination period, the samples did not degrade at all. The average weight of the measured samples (mg) ± SD can be found in Table 2. In the first column, the samples’ weight can be seen without diclofenac after the printing, and in the others, the measured weight in the 1st, 2nd, 4th, 6th, and 8th weeks.

### 2.4. Characterization

#### 2.4.1. Thermogravimetric (TG) and Heatflow (Differential Scanning Analyis, DSC) Analysis

**Figure 3:**
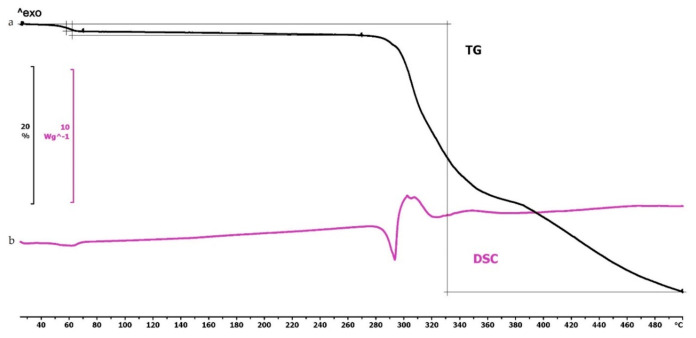
Thermogravimetric and heatflow analysis of the diclofenac sodium powder: (**a**) Thermogravimetric (TG) curve; (**b**) heatflow (Differential Scanning Analyis, DSC) curve. Thermal behavior was analyzed between 25 and 500 °C.

**Figure 4:**
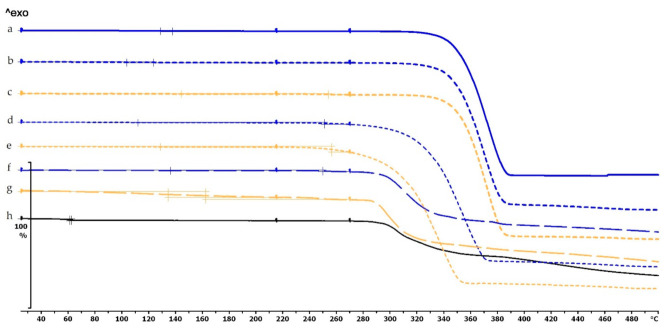
Thermogravimetric curves of PLA and antibacterial PLA (Anti) filaments and samples without and with diclofenac: (**a**) PLA filament; (**b**) PLA_19_0 without diclofenac; (**c**) Anti_19_0 without diclofenac; (**d**) PLA_19_0 polymer frame after the API was poured out; (**e**) Anti_19_0 polymer frame after the API was poured out; (**f**) diclofenac powder from sample (**d**); (**g**) diclofenac powder from sample (**e**); (**h**) diclofenac sodium powder. The thermal behavior was analyzed between 25 and 500 °C.

**Figure 5:**
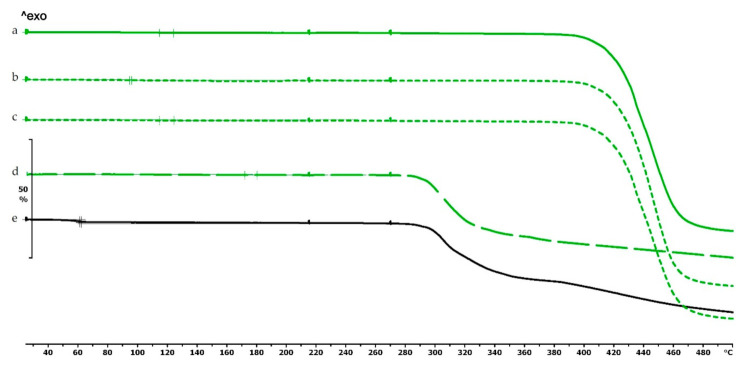
Thermogravimetric curves of PETG filament and samples without and with diclofenac: (**a**) PETG filament; (**b**) PETG_19_0 without diclofenac; (**c**) PETG_19_0 polymer frame after the active pharmaceutical ingredients (API) was poured out; (**d**) diclofenac powder from sample (**c**); (**e**) diclofenac sodium powder. Thermal behavior was analyzed between 25 and 500 °C.

**Figure 6:**
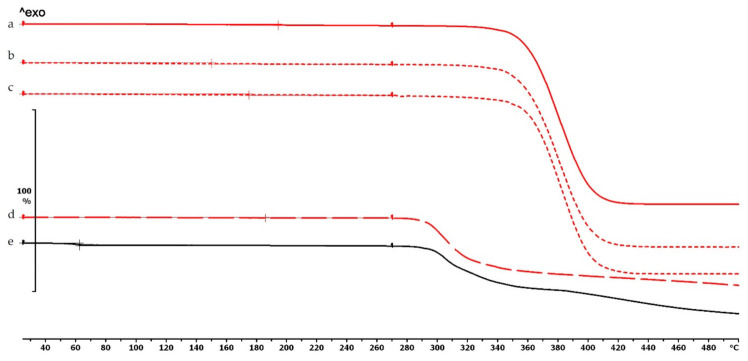
Thermogravimetric curves of PMMA filament and samples without and with diclofenac: (**a**) PMMA filament; (**b**) PMMA_19_0 without diclofenac; (**c**) PMMA_19_0 polymer frame after the API was poured out; (**d**) diclofenac powder from sample (**c**); (**e**) diclofenac sodium powder. Thermal behavior was analyzed between 25 and 500 °C.

The TG curve of diclofenac sodium showed thermal stability until 280 °C, so we can state that the API used is stable at the applied printing temperatures. Below 100 °C, a less than 1% mass loss was observed, which was probably due to absorbed water evaporation from the surface of the API. In the diclofenac powder samples poured out from the polymeric frames, this did not occur (Figures 4f,g, 5d and 6d, respectively). Based on the TG curve of the API, it is very likely that melting was associated with thermal decomposition. The API decomposition was around 39% until 500 °C. The DSC curve showed an endothermic peak at 289.97 °C, which was immediately followed by an exothermic peak where the diclofenac decomposed (Figure 3).

The thermogravimetric analysis of the PLA, antibacterial PLA, PETG, and PMMA samples was performed on filaments, polymeric samples without diclofenac, and polymeric samples with diclofenac, but the API was poured out before the TG analysis (polymer frame). In these graphs, the diclofenac TG curve and the diclofenac powder from the polymer frames can be seen (Figure 4, Figure 5 and Figure 6). The curves proved that all the APIs and the filaments were stable at the printing temperature of the experiment; no sign of chemical decompositions or changes was detected below 270 °C. All four polymers can be considered thermally stable, but the polymers can be grouped based on the temperature range: PLA and antibacterial PLA had a stable maximum of 280 °C, PETG was stable until 400 °C, and PMMA was stable until 340 °C. We can state that at the applied printing temperatures (PLA—215 °C, PETG—250 °C, and PMMA—270 °C), all the polymers were stable.

In the case of PLA and antibacterial PLA polymer frames (Figure 4d,e), the thermal stability was smaller at 50 °C in comparison with the samples without diclofenac (Figure 4b,c); these samples can still be considered stable because the printing temperature was 215 °C. The PETG and PMMA polymer samples did not show a similar alteration; the thermal stabilities were not altered with or without diclofenac sodium (Figure 5 and Figure 6).

#### 2.4.2. Contact Angle Measurement

**Figure 7:**
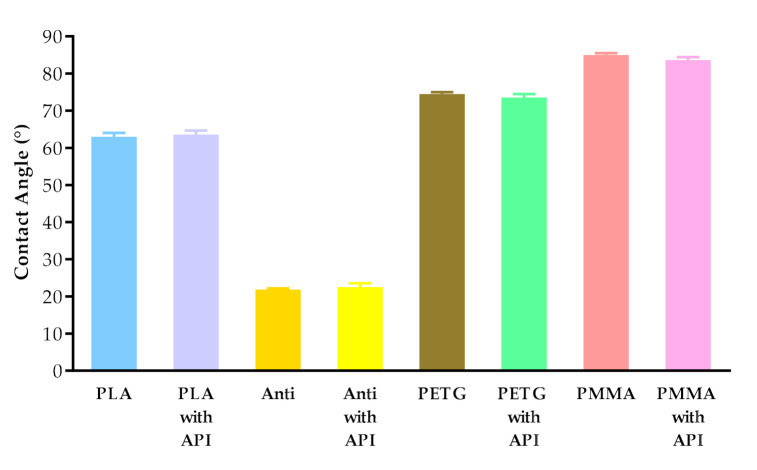
Contact angle values of PLA, antibacterial PLA (Anti), PETG, and PMMA samples after printing without and with diclofenac sodium (with API). Data are expressed as means ± SD. Experiments were performed in triplicate, *n* = 3.

The contact angle values are given in Figure 7. The contact angle values were measured on four types of polymer (PLA, PLA antibacterial, PETG, and PMMA), without and with diclofenac sodium. The different polymers had different contact angle values: PLA around 63°, antibacterial PLA around 22°, PETG around 74°, and PMMA around 84°. Interestingly, the contact angle values of antibacterial PLA filaments decreased nearly to one-third compared to the PLA contact angle values due to the antibacterial nanoparticles in the filament.

#### 2.4.3. Scanning Electron Microscopy

**Figure 8:**
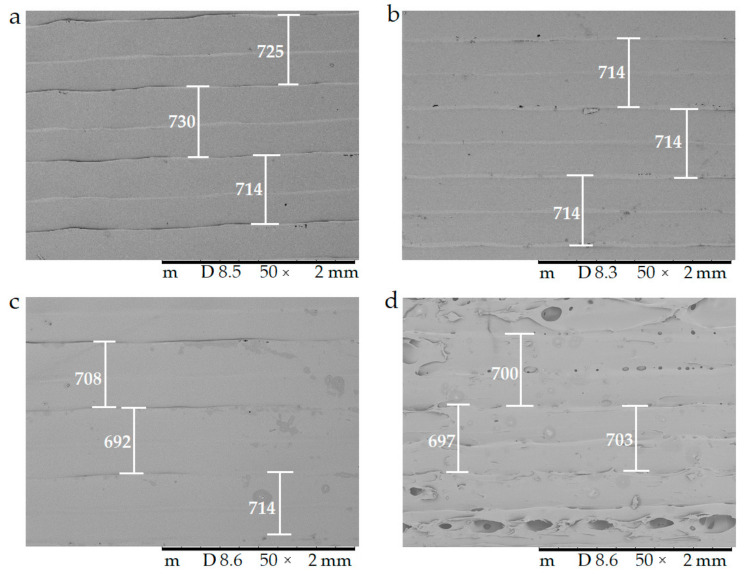
The surface morphology of the 3D-printed samples was characterized using scanning electron microscopy (SEM). Magnification was 50×, the scale bar is in µm, and the layer thickness is labeled with the white bars. (**a**) Polylactic acid PLA_22_ 0; (**b**) antibacterial polylactic acid (Anti_22_0); (**c**) polyethylene terephthalate glycol (PETG_22_0); (**d**) poly(methyl methacrylate) (PMMA_22_0).

The surfaces of the samples were examined with scanning electron microscopy. The SEM images (Figure 8) of the PLA, Antibacterial PLA, PETG, and PMMA samples were determined at 50× magnification. The surface adequateness was measured, and the layers were properly structured on the surface of the 3D-printed samples. In the figure, the distance between two printed filaments next to each other is labeled with white bars. In the case of PLA, the average was 713.67 µm (±17.72); for antibacterial PLA, it was 712.86 µm (±2.26); for PETG, it was 704.33 µm (±12.07), and for PMMA, it was 700.16 µm (±7.73). PLA, antibacterial PLA, and PETG had flat but PMMA had a stepped surface, with some small deviations from planarity.

**Figure 9:**
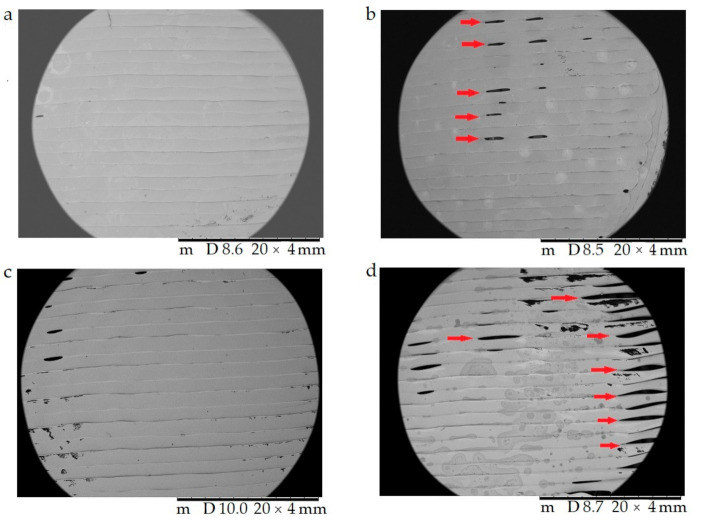
The surface morphology and pore structure of the 3D-printed samples were characterized using scanning electron microscopy (SEM) right after the 3D printing (**a**,**c**) and after the dissolution test (**b**,**d**). Magnification was 20×. (**a**) Antibacterial polylactic acid (Anti_22_0) sample after the 3D printing; (**b**) Anti_22_0 after the dissolution test; (**c**) polyethyelene terephthalate glycol (PETG_22_0) sample after the 3D printing; (**d**) PETG_22_0 after the dissolution test. The pores formed after the dissolution test (**b**,**d**) are labeled with red arrows.

In Figure 9, the surface morphology and pore structure of the 3D-printed samples are compared right after the 3D printing (a,c) and after the dissolution test (b,d) with 20× magnification. The red arrows in (b) and (d) show the developed pores after the dissolution test, which supported our assumption that API liberation happened with diffusion through the API dissolving holes.

#### 2.4.4. Microcomputed Tomography (MicroCT)

**Figure 10:**
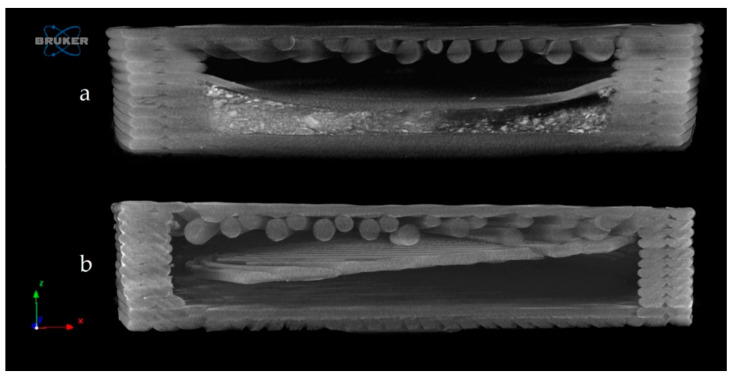
Reconstructed microcomputed tomography (microCT) image from a vertical cut of Anti_16_0 sample before (**a**) and after (**b**) dissolution test. Image pixel size is 5 μm.

The samples were examined by microcomputed tomography (microCT) before and after the dissolution test to determine their morphology. Figure 10 represents the Anti_16_0 sample before (a) and after (b) the dissolution test. The localization of the diclofenac sodium is clearly seen before the dissolution test. The dissolution test did not affect the location of the lateral filament layers.

**Figure 11:**
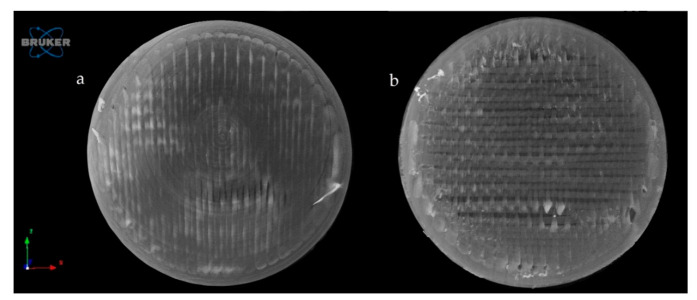
Reconstructed microCT image from the upper surface of PLA_16_0 sample before (**a**) and after (**b**) dissolution test. Image pixel size is 5 μm.

Figure 11 represents the upper surface of the PLA_16_0 sample before (a) and after (b) dissolution. It can be clearly seen that pores formed on the surface of the dissolved sample.

The exact difference was calculated from three parallel sliced surfaces of the 5 µm image pixel size. The percentage of the filament infill was examined. The PLA_16_0 sample before dissolution showed 94.06% area, and after dissolution, it was 87.95%. There were 6.11% more pores on the surface of the sample after dissolution.

#### 2.4.5. Raman Spectroscopy

**Figure 12:**
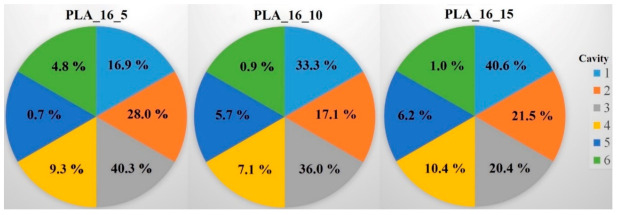
Distribution of diclofenac sodium salt in percentage within the six cavities (1‒6) for the PLA_16_5, PLA_16_10, and PLA_16_15 samples. In PLA_16_5 and PLA_16_10, the most API was in cavity 3; in PLA_16_15, the most was in cavity 1, but 75% of the whole diclofenac amount was in cavities 1‒3 in all samples. The standard deviation results can be found in Table 3.

Raman spectroscopy was performed on a Wasatch Photonics WP-785-R-SR_l-50 equipment (Morrisville, NC, USA) to determine the distribution of the diclofenac sodium salt within the six cavities of the 5%, 10%, and 15% infill samples. The detector was a charge-coupled device (CCD), the detector temperature was 10 °C, the laser beam wavelength was 785 nm, the integration time was 500 ms, and the scan time was 32 s. The data were analyzed in Unscrambler X version 10.5.1 software (Camo Analytics, Montclair, NJ, USA). For these measurements, polylactic acid samples with 16 mm diameter and 0%, 5%, 10%, or 15% infill were used. The sample with 0% infill had only one cavity, so in this case, the whole amount of API was determined. For the PLA_16_5, PLA_16_10, and PLA_16_15 samples, the diclofenac amount in every cavity was determined with the use of the calibration curve, and the API amount in milligrams is given in Table 3. All samples contained approximately 30 mg of diclofenac sodium salt; these results were in accordance with the content uniformity measurements (Table 1). In Figure 12, the samples with six cavities (5%, 10%, and 15% infill), and the amount of API in every cavity was labeled. Based on these results, around 75% of the whole API amount was in cavities 1, 2, and 3 in sample PLA_16_5, PLA_16_10, and PLA_16_15.

### 2.5. In Vitro Dissolution Test

The dissolution profiles of the samples were determined by a USP type I in vitro dissolution apparatus to gain information about the effect of the polymers, diameters, and infill percentages on the dissolution profiles. The diffusion rate (µg·mL^−1^·h^−1^) and flux (µg·cm^−2^·h^−1^) of the samples were also calculated (Table 4). The diffusion rate of the PLA, antibacterial PLA, PETG, and PMMA samples was calculated for two time intervals, 0‒2 h and 2‒24 h, from the concentration and time data. Flux parameters were also calculated by the concentration data, time data, and the surface area of the samples (for 16 mm, 5.23 cm^2^; for 19 mm, 7.10 cm^2^; and for 22 mm, 9.3 cm^2^). These results represent the dissolved API amount through the surface area. Within the first 2 h, our samples can be described with different diffusion rate results, and between 2 and 24 h, a plateau phase can be seen. 

The dissolved API amount (%) at every sampling time and the standard deviation (±SD) results can be found in Table A1, but the dissolved API amount (%) at 2 h and 24 h can also be found in Table 4. At 2 h, the dissolved API amount varied from 16% to 97%. The dissolution from the PMMA_19_0 sample stopped after 2 h. At 24 h, we found that the dissolved API amount varied between 50% and 90% depending on the polymer, the diameter, and the infill percentage. PETG_19_0 and PMMA_22_0 showed the highest API release with values of 99.49% and 99.48%, while for PLA_22_10, the value was only 51.26% at 24 h.

Every sample was analyzed by an unpaired *t*-test for the 2 h and 24 h dissolved API amounts. The F test was nonsignificant in all cases. The results can be found in Table 4. We found the results statistically nonsignificant in the case of the PETG_16_0 and PMMA_19_0 samples because the API dissolution already reached the plateau phase by the second hourly measurement point based on the dissolved API amount results.

**Figure 13:**
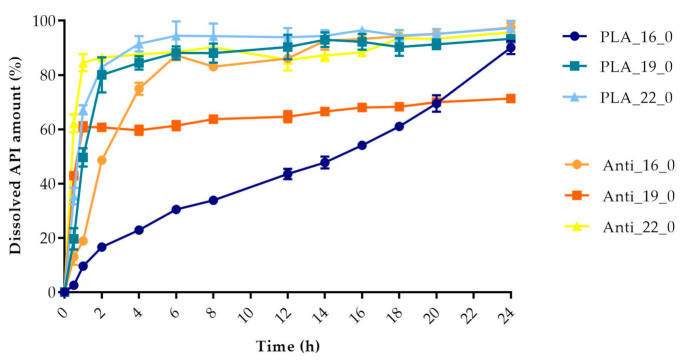
Dissolved API amount (%) versus time (h) for polylactic acid (PLA) and antibacterial PLA samples; mean ± SD, *n* = 3.

**Figure 14:**
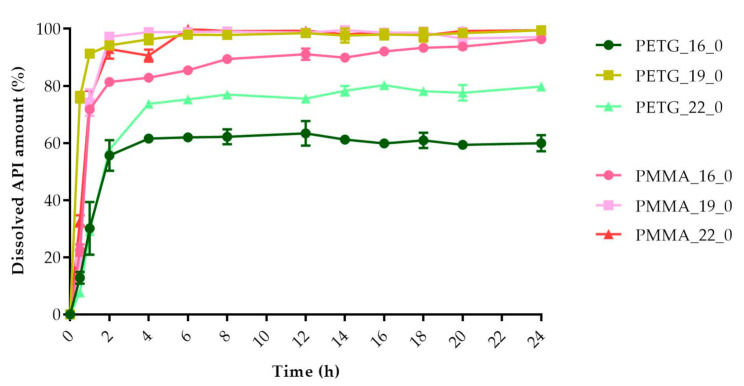
Dissolved API amount (%) versus time (h) for polyethylene terephthalate glycol (PETG) and poly-(methyl methacrylate) PMMA samples; mean ± SD, *n* = 3.

**Figure 15:**
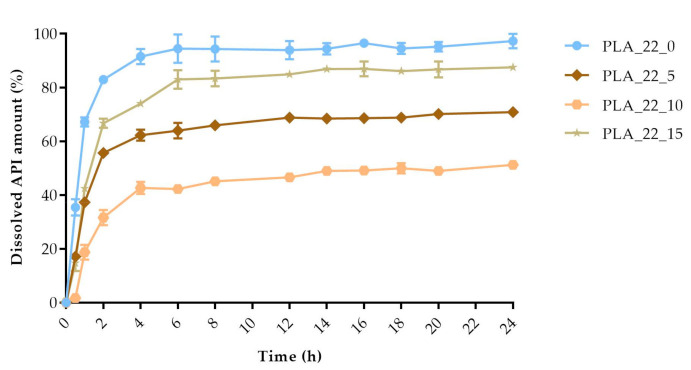
The polylactic acid (PLA) samples with 22 mm diameter and different infill percentages: 0%, 5%, 10%, and 15%. The dissolved API amount (%) was plotted against time (h), mean ± SD, *n* = 3.

The dissolved API amount (%) was plotted against the time (h) for the polylactic acid (PLA), antibacterial PLA (Figure 13), polyethylene terephthalate glycol (PETG), and poly(methyl methacrylate) (PMMA) samples (Figure 14). The dissolution curve of PLA samples with different infill percentages (PLA_22_0, PLA_22_5, PLA_22_10, and PLA_22_15) can be found in Figure 15. The results are means ± SD, *n* = 3.

Pairwise comparison results of the dissolution profiles can be found in Table A2: the f1 value of the difference factor calculation and f2 value of the similarity value calculation. We found that all of our sample dissolution profile can be considered nonsimilar. Based on this comparison, all samples had different dissolution curves.

Drug release data were fitted to zero-order and first-order models (Table 5). Determination coefficients were used to determine the best fit. The PLA_16_0 sample fitted to the zero-order model, which confirms the linear curve shape (Figure 13). The Anti_16_0 sample showed first-order kinetics, with a correlation coefficient higher than 0.93. The calculations revealed that other samples can be fitted neither to the zero-order nor the first-order model, since the correlation coefficients were all smaller than 0.80 if we compared the results from 0 to 24 h. The dissolved API amounts were fitted to the same kinetic models but only at 0‒2 h. PLA_19_0, PLA_22_0, PLA_22_5, PLA_22_10, PLA_22_15, PMMA_16_0, PMMA_19_0, and PMMA_22_0 can be considered zero-order kinetics in the first 2 h, while PETG_16_0 and PETG_22_0 fitted more to the first-order kinetic model at 0‒2 h. The Anti_19_0, Anti_22_0, and PETG_19_0 samples (labeled with * in the table) were examined based on their dissolution curves. First, we determined the last time point before the curve reached the plateau phase and the kinetic models were fitted only before that time point. Anti_19_0 and PETG_19_0 until 30 min and Anti_22_0 until 45 min were fitted to a first-order kinetic model.

**Figure 16:**
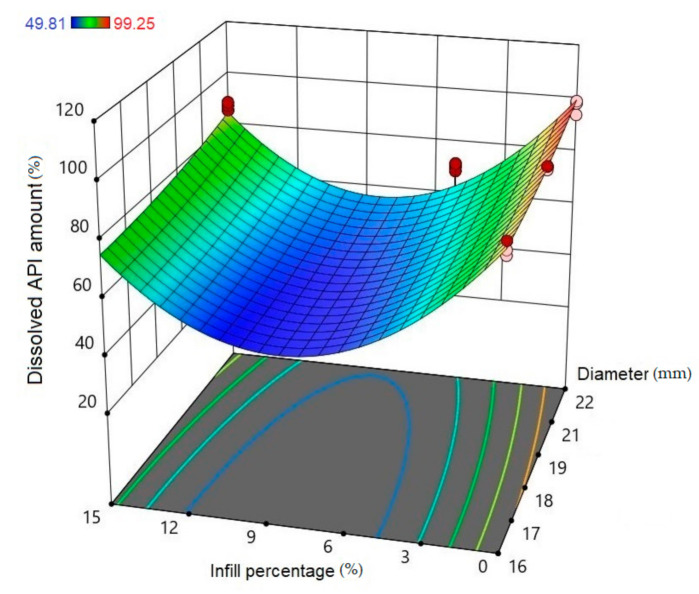
Three-dimensional (3D) diagram of the polylactic acid (PLA) samples after 24-h dissolution. The diagram was prepared with Design-Expert^®^ software. The *X*-axis represents the infill percentage (%), the *Y*-axis represents the dissolved API amount (%), and the *Z*-axis represents the sample diameter (mm). Red circles represent the design points above the predicted value, and pink circles represent the design points under the predicted value. In the top left corner, the dissolved API amount (%) can be seen, where the dissolved API is labeled with a different color. Blue represents the dissolution starting at 49.81% and red ends at 99.25%.

**Figure 17:**
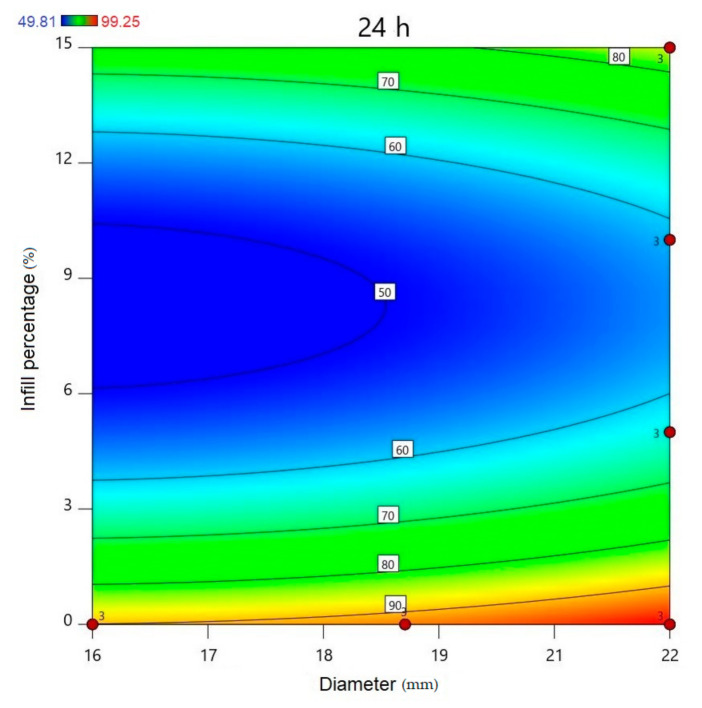
All polylactic acid (PLA) samples after 24-h dissolution; the infill percentage (%) is plotted against the diameter (mm). red circles represent the design points above the predicted value, and “3” means the sample amount was above the predicted value. The numbers in the squares represent the dissolved API amount in percentage.

Using Design-Expert^®^ software (Stat-Ease Int., Minneapolis, MN, USA), historical statistical data analysis was performed to determine the effects of the diameter and infill percentage on the dissolution profiles. In Figure 16, the three-dimensional diagram of the PLA samples in case of the 24 h dissolution time can be seen. The three axes represent the infill percentage (%), the dissolved API amount (%), and the sample diameter (mm). The most API was dissolved from the samples in the case of 0% infill. The higher the diameter of the sample, the higher the dissolved API amount. Interestingly, the infill percentage was better at 0% and 15% infill, but the lowest predicted value was around 9% infill. The 2D diagram of the same results can be seen in Figure 17.

The diameter and infill percentage have a similar effect to what was expected. The dissolution was faster with the increase in the diameter due to the larger surface area and the formation of more API-dissolving pores. The API dissolution from the samples can be slowed down with the alteration of the infill percentage from 0% to higher amounts, e.g., 5%, 10%, or 15%.

### 2.6. MTT Assay

A prolonged cell viability test was performed to gain information about the cytocompatibility of the 3D-printed samples. The samples were incubated in the cell culture medium for 4, 8, and 12 days, and the monolayer formed by Caco-2 cells was treated with this medium to determine if any kind of xenobiotic was dissolved from the sample. This method differed from the original MTT assay because the inhibitory concentration (IC_50_) was not measured, but the cell viability was calculated in comparison with the untreated, negative control (DMME medium). This method was harmonized with the ISO standard.

**Figure 18:**
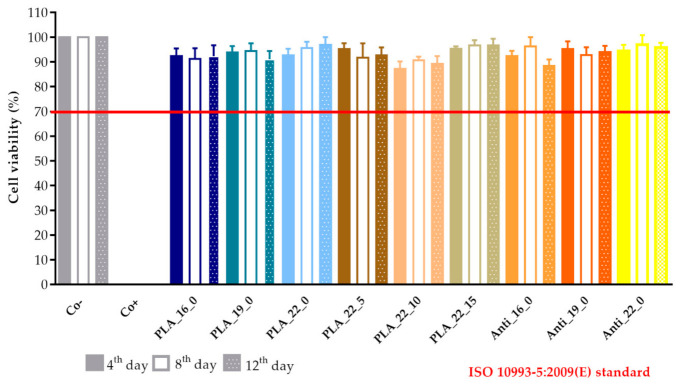
The prolonged cytotoxicity of the polylactic acid (PLA) and antibacterial PLA (Anti) samples with different diameters (16, 19, or 22 mm) and infill percentages (0%, 5%, 10%, or 15%). MTT ((3-(4,5-dimethylthiazol-2-yl)-2,5-diphenyl-2*H*-tetrazolium bromide dye) cell viability tests were performed on days 4, 8, and 12. Cell viability was expressed as the percentage of untreated control (Co−). As a positive control (Co+), Triton X-100 (10% *w/v*) was used. Data were expressed as means of three independent experiments ± SD.

**Figure 19:**
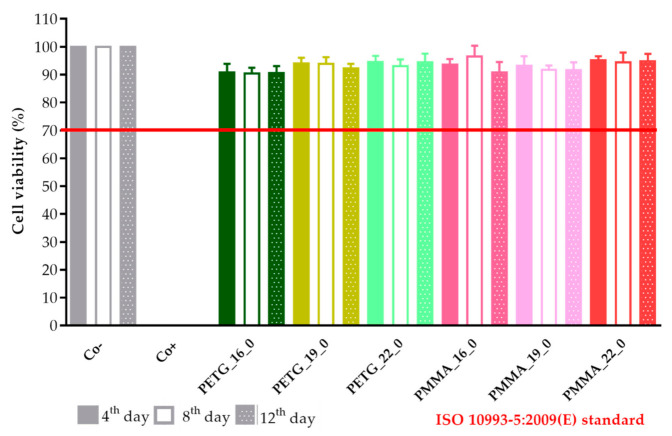
The prolonged cytotoxicity of the polyethylene terephthalate glycol (PETG) and poly(methyl methacrylate) (PMMA) samples with 16, 19, or 22 mm diameter and 0% infill on CaCo-2 cells determined by an MTT cell viability test on days 4, 8, and 12. Cell viability was expressed as the percentage of the untreated control (Co−) in the case of the PETG and PMMA samples. As a positive control (Co+), Triton X-100 (10% *w/v*) was used. Data were expressed as the means of three independent experiments ± SD.

The results were expressed as the percentage of negative or untreated control (Co−). (Figure 18 and Figure 19) As a positive control (Co+), Triton X-100 (10% *w/v*) solubilizing agent was used, which has significant differences from the other examined samples. Based on the ISO 10993-5:2009(E) standard, if the relative cell viability was higher than 70% in comparison with the control group (100%), the materials could be considered noncytotoxic [41]. According to this regulation, all 3D-printed PLA, antibacterial PLA, PETG, and PMMA samples qualified as cytocompatible.

## 3. Discussion

In our study, different samples of PLA, antibacterial PLA, PETG, and PMMA polymers with 16, 19, or 22 mm diameters and 0%, 5%, 10%, or 15% infill were printed using FDM technology to manufacture an implantable drug delivery system that enables fast manufacturing from commercially available filaments. The reason for these investigations was to certify the applicability of the FDM method in the field of personalized medication in surgeries. Some authors reported that personalized medication was an excellent response to the need for appropriate and adequate healthcare. The APIs can be delivered at the right dose and at the right time [46].

Three-dimensional (3D) printing significantly speeds up the design cycle, both in the development and in the industrial manufacturing [47]. A research group described that the physical and chemical properties of implant materials greatly influence the utility of the implant—for example, the release profile or the biodegradation/integrity [48]. Different designs can be manufactured. A bullet-shaped implantable system with a porous surface was planned to provide controlled cytoxan release [49]. In a study, hollow 3D-printed implants with similar dimensions were prepared. In this case, the implant manufacturing required a lot of steps and three different polymers. First, the main frame was printed from PLA with a different amount of polyvinyl alcohol polymer window. Ibuprofen, the model API, was directly packed inside. Finally, the samples were coated with a polycaprolactone formulation [50].

Tailoring the performance of the 3D printing process to the infilling of the API may be difficult. Our research group developed an easy method to fill the samples with the API. First, a two-layer cover is printed for each sample in advance, which is then placed on the top of the API-containing lower part; then, the 3D printing is finished with the printing of the upper part. The degradation of the API due to the high printing temperature (210‒270 °C) and the loss of API due to ventilation may also be eliminated [51]. The APIs’ weight loss was around 5% after the FDM printing from polyvinyl alcohol filament. In the pharmaceutical industry, only a maximum of 1% API deviation can be accepted [52]. In a study, the filament contained 50% of the API, but it was stated that by increasing the dispersal of the API content, the properties of the filaments for 3D printing could be altered, which would have effects on the printability and the product properties. The interplay through the printing is not well understood yet, and further investigations are required [53]. In our work, the developed FDM process is applicable because the API amount and stability are not affected. As it is an implantable drug delivery system, API can be directly applied to the immediately printed implants and the drug delivery system may be applied during surgical interventions.

The TGA results of diclofenac sodium confirmed that API was stable up to 280 °C, and our samples with diclofenac were stable after the 3D printing. Based on our results, no chemical decompositions or changes were detected below 270 °C in the polymers. All polymers can be considered thermally stable, which enables the filament sterilization. The sterilized filaments can be directly printed in the operating room [54].

Contact angle results showed that PLA, antibacterial PLA, PETG, and PMMA had different wettability, which can affect the samples’ rejection in the human body [55]. In the case of antibacterial PLA, which is a PLA modification, the contact angle results decreased, but based on research, the surface hydrophilicity increased. The low contact angle indicated that the interaction of material and water molecules was high, resulting in the high hydrophilicity of the surfaces [56].

The impacts of the polymer and the geometry (diameter and infill percentage) on the dissolution profile of diclofenac sodium were considered. It is expected that if the diameter increases, the number of pores on the lower and upper surfaces will increase as well, and the dissolution of the API will be faster. In the PLA_16_0, PLA_19_0, and PLA_22_0 samples, with the increase in diameter, the dissolution was faster because of the larger surface area. If the infill percentage increases within the structure, it hinders the dissolution. In our study, we found that the sample with 0% infill had the fastest dissolution due to the lack of contact points, but the increase in the infill percentage (5%, 10%, or 15%) did not constantly influence the dissolution. The horizontal surfaces manufactured by FDM-based 3D printers did not hermetically seal the inner volume and may affect the diffusion of APIs [57]. These two parameters influence the dissolution in an expected way. The polymer type does not have a significant impact on the dissolution profiles, but the results show that PMMA has the highest drug release after 24 h.

Based on a study, Raman spectroscopy can be used in different ways throughout the characterization. The distribution of different APIs can be determined, and the particle size and shape can be examined [37]. We found that around 70% of the whole API amount was in cavities 1, 2, and 3 in samples PLA_16_5, PLA_16_10, and PLA_16_15. Based on the results, all samples contain 30 mg diclofenac sodium salt; these results are in accordance with the content uniformity measurements.

The samples’ surfaces were examined via scanning electron microscopy (SEM) to determine the surface morphology. After 3D printing, the samples’ surfaces looked flattened, without pores, but after the dissolution tests, pores had formed on the surface, so the dissolution can be described as a free diffusion. The microCT results confirmed the SEM results and the pore amount was determined; a 6.11% increase was found.

It was determined that oligomers and solubilized drug molecules are able to diffuse through the polymeric reservoir system [58]. The dissolution of drugs from drug delivery systems depends on various physical and chemical rules, which results in difficulties in ascribing proper mathematical models to the processes occurring [59], so our samples were examined with first-order and zero-order kinetics at different time intervals [60,61]. In our research, the PLA_16_0, PETG_16_0, and PETG_22_0 samples could be described with a zero-order kinetic, but all the other samples presented first-order kinetics. The findings suggested that changes in the implant microstructure could significantly alter the drug release. Therefore, researchers have to design implants that reduce these changes, so a correlation between in vitro/in vivo drug releases profiles can be achieved [62].

As PLA and antibacterial PLA are biodegradable polymers, the degradation was investigated at pH = 7.4, 37 °C by weight measurement, but within these eight weeks, the samples did not degrade at all. Based on a study in aqueous solutions, the hydrolytic degradation depends on different factors [63]. It is well known that the pKa of lactic acid is 3.84 [64], and the hydrolysis is fast at a low or high pH [65]. Some authors investigated the degradation at varying pH (1.5, 4.5, or 7.4) at 65 °C. It was found that the degradation rate depends on the molecular weight, the temperature, and the pH [66]. Based on these studies, we can state that our samples did not degrade within eight weeks at 37 °C and pH = 7.4.

The in vitro cytotoxicity profile of the samples was determined with a long-term MTT assay. The method was harmonized with the ISO 10,993 standard but with a shorter incubation period [41]. The sterile samples were stored in Dulbecco’s Modified Eagle’s Medium (DMEM) at 37 °C, and the potentially dissolved xenobiotics were measured by MTT assay on days 4, 8, and 12 [40]. The in vitro cytotoxicity method is a compulsory test as part of the biocompatibility measurements. Cytotoxicity can be a first filter through the determination of biocompatibility and can be a good predictor of the in vivo results [67]. These measurements can be performed on different cell lines such as human keratinocyte cell line (HaCaT), mammalian cell line (HeLa), human pancreatic carcinoma (PaCa-2), or colon carcinoma (CaCo-2) depending on the dosage form and the place of application. Our experiments were performed on the Caco-2 cell line because it is still the gold standard for cytotoxicity examinations [68,69].

However, several assays can be performed to determine cytotoxicity prior to reaching complete integrity, such as end-point or noninvasive cell viability assays (LDH test, real-time cell electronic sensing assay (RT-CES), etc.), but the MTT assay, as a rapid colorimetric method, is still applied to determine the in vitro cytotoxicity [70]. It was revealed that the MTT dye alteration to formazan salt depends on the cells’ metabolic rate and number of mitochondria [39]. In spite of this limitation of the MTT assay, it is still the most reliable and quickest method for the assessment of cytocompatibility [71]. Based on the prolonged MTT assay, results were in accordance with the ISO 10993:5 standard, and all our samples can be considered cytocompatible.

In conclusion, 12 3D-printed samples with different diameters, infill percentages, and four types of polymers were printed adequately in our work. This special design ensures that all kinds of API can be utilized during printing without heat damage or API loss, confirmed by TG and Raman spectroscopy results. The diameter and infill percentage have the expected effect on the dissolution profile. SEM and microCT images confirmed that the dissolution takes place through pores on the surface by diffusion. Based on the MTT assay results, our samples can be considered cytocompatible. Contact angle values determined that antibacterial PLA had the most hydrophilic surface. Based on the overall project, PLA and antibacterial PLA have been suggested for the preparation of an implantable drug delivery system.

## 4. Materials and Methods

### 4.1. Materials

#### 4.1.1. Used Polymer Filaments

For the 3D printing process, we purchased polylactic acid (PLA), antibacterial polylactic acid (Anti PLA), polyethylene terephthalate glycol (PETG), and poly(methyl methacrylate) (PMMA) polymers from Philament Kft. (Philament Kft., Miskolc, Hungary). The filament diameters were 1.75 mm. The properties of the commercially available filament and the 3D-printed filament are compared in Table 6 and Table 7 below. The data were provided by Philament Kft (Philament Kft., Miskolc, Hungary).

#### 4.1.2. Diclofenac Sodium as a Model API

Diclofenac sodium salt was purchased from Tokyo Chemical Industry Co. (Tokyo, Japan).

### 4.2. Methods

#### 4.2.1. Design of the Drug Reservoirs and Printing of the Samples

**Figure 20:**
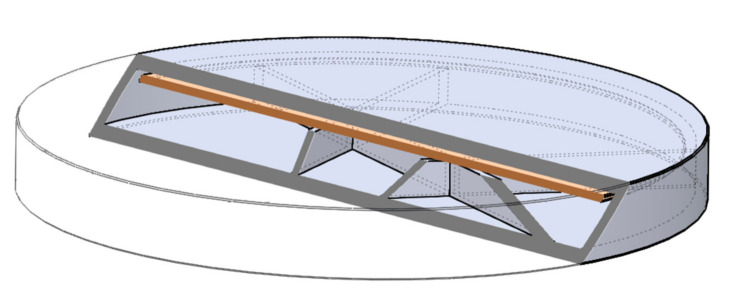
Three-dimensional (3D) sliced structure of the printed sample with 5% infill. The brown layer represents the two-layered extra cover.

The digital models of the samples were designed using SolidWorks (Dessault Systèmes, Vélizy-Villacoublay, France), which is a solid modeling computer-aided design and computer-aided engineering software. Exporting the design into an.stl (standard tessellation language) file makes it customizable, which provides flexibility for the user and is preferred by 3D designers as well [72]. The structure of the designed sample can be found below (Figure 20).

The shape was based on a cylinder, but the edges were chamfered because it was more beneficial for the 3D printing process. Each tablet was 2.4 mm tall, but three different diameters were used: 16, 19, or 22 mm. Since API dissolution mainly happens through the top and bottom surface of the sample, diameters were computed to have an increase of 150% or 200% in area. All the other geometric and additional properties of the samples were determined in the slicing software [73].

Batches of 10 implantable drug delivery systems were printed on a Prusa i3 MK2 printer (Prague, Czech Republic). The inner structure was designed by the Slicer Prusa Edition software and the printing parameters such as infill pattern (cubic) and percentage (0%, 5%, 10%, or 15%), number of shells (1), and number of top and bottom layers (2) was adjusted. The parameters of the 3D printing process are summarized below in Table 8 [42].

#### 4.2.2. Content Uniformity and Weight Variation

Before the experiments, 10 samples from each formulation were individually weighed and the calculated average with standard deviation gave the weight variation. Then, the samples were crushed for content uniformity determination. The samples were separately dispersed in 100 mL of distilled water and stirred for 60 min. The drug amount was determined using UV spectrophotometry with ThemoScientific™ Multiskan™ GO Microplate Spectrophotometer (Waltham, MA, USA) at a wavelength of 276 nm [74].

#### 4.2.3. PLA Degradation

In vitro degradation of PLA samples was performed. The samples were incubated in simulated body fluid (SBF) at 37 °C with stirring at 10 rpm for up to eight weeks. (Heidolph Incubator 1000 and Polymax 1040, Schwabach, Germany). The SBF solution was prepared by dissolving 0.353 g NaHCO_3_, 0.224 g KCl, 7.995 g NaCl, 0.305 g MgCl_2_·6 H_2_O, 0.228 g K_2_HPO_4_·3 H_2_O, 0.071 g Na_2_SO_4_ and 0.227 g CaCl_2_ into 1 L of distilled water [75]. The pH was buffered at 7.4 with Tris-HCl. For the degradation test, three samples in parallel were immersed in 20 mL of SBF solution. At weekly intervals, the samples were taken out, washed with distilled water, and dried in an incubator at 60 °C. The weight was measured with a MettlerToledo AX105 DeltaRange Analytical Balance (Columbus, OH, USA) and the average was calculated. The incubation medium was replaced with fresh SBF solution weekly [35].

#### 4.2.4. Characterization

##### Thermogravimetric (TG) and Heatflow (Differential Scanning Analyis, DSC) Analysis

The thermogravimetric (TG) and heatflow (Differential Scanning Analyis, DSC) analyses of the samples were carried out with a Mettler-Toledo TGA/DSC1 instrument (Mettler-Toledo GmbH, Urdorf, Switzerland). The samples were placed in a closed aluminum crucible at a volume of 40 µL. The temperature range was 25–500 °C, and the heating rate was 10 °C/min. Nitrogen atmosphere was used (Cell gas: 50 mL/min, method gas: 70 mL/min). Evaluation of the TG/DSC curves was performed with STAR^e^ software (Mettler-Toledo GmbH, Budapest, Hungary) [76].

##### Contact Angle

Contact angle measurements were performed on a Kruss goniometer (Kruss, Hamburg, Germany). A total of 10 µL of deionized water was deposited on the surface of the samples, and the droplet shape was imaged. The contact angle was determined using the internal Kruss software. Measurements were carried out a minimum of ten times for each sample, and the results were the average of the measurements [56].

##### Scanning Electron Microscopy (SEM)

Scanning electron microscopy images were taken with a Hitachi Tabletop Microscope 3030 (TM3030) (Hitachi, Tokyo, Japan). Before SEM observation, a gold-sputtered coating was deposited on the surface to avoid the charging and melting of the samples. The measurement requires a vacuum and low accelerating voltage. In this TM3030 microscope, the detector used is an energy-dispersive detector (EDS). The images were taken at different magnifications [77].

##### Microcomputed Tomography (MicroCT)

A SkyScan 1272 (Bruker, Kontich, Belgium) compact desktop microcomputed tomography (microCT) system was used for the measurement. Scanning parameters were as follows: image pixel size, 5 μm; matrix size, 2688 × 4032 (rows × columns); source voltage = 50 kV; source current = 200 μA; rotation step (deg) = 0.200. Flat field correction and geometrical correction were used. After scanning, SkyScan NRecon software (version 2.0.4.2) was used to reconstruct cross-section images from tomography projection images. Post-alignment, beam hardening correction, ring artifact correction, and smoothing were done. The output formats were DICOM and BPM images. For 3D image visualization, CTwox software (Bruker, Kontich, Belgium) was used [77].

The exact difference was calculated by ImageJ software (Madison, WI, USA). For the measurement, three parallel sliced surfaces of 5 µm image pixel size were examined. The original microCT images were transformed to 8-bit grayscale images. This step converted each pixel’s color information into a brightness measurement. In the threshold dialog window, we could change all pixels into black and white depending on the adjusted range. Then, we marked the surface of the sample, and finally, the software automatically evaluated the percentage of the filament infill [78].

##### Raman Spectroscopy

The API amounts of the PLA_16_0, PLA_16_5, PLA_16_10, and PLA_16_15 samples were determined by Raman spectroscopy. The PLA_16_0 sample had one cavity as the infill percentage was 0%, but the other samples with 5%, 10%, and 15% infill rates formed six inside cavities. First, a hole was drilled on the surface of one cavity with a small-diameter (<0.5 cm) drill bit. Then, the gained API was dissolved in 4.4 mL of 96% ethanol. For the determination of the API amount, a seven-point calibration curve was prepared in the concentration range of 0.1‒15 mg/mL. Raman spectra were determined in a quartz cuvette with a 1-cm optical pathway. The spectra were analyzed with multivariate regression modeling based on the partial least squares (PLS) method in CAMO Unscrambler X software (Oslo, Norway). The diclofenac amount in every cavity was determined from the calibration curve [79].

#### 4.2.5. In Vitro Dissolution Test

The dissolution test was carried out using a USP Type I Erweka DT 800 dissolution apparatus (Erweka GmbH, Langen, Germany) with an automatic sampling system, Ismatec IPC High-Precision Multichannel Dispenser (Ismatec GmbH, Wertheim, Germany). As a dissolution medium, 900 mL of phosphate buffer (1.0 M, pH 7.4) was used (Sigma Aldrich, Saint Quentin Fallavier, France). The rotation speed was set to 100 rpm and the temperature was set to 37 °C. All samples were placed in the basket. Samples of 3 mL were collected at 0.083, 0.25, 0.5, 0.75, 1, 2, 4, 6, 8, 12, 14, 16, 18, 20, and 24 h using the autosampler [17]. The concentration of the release drug was determined by UV spectrophotometry with Thermo Scientific™ Multiskan™ GO Microplate Spectrophotometer at a wavelength of 276 nm. All samples at all measurement points were put in 96-well UV-Star^®^ microplates (Greiner Bio-One Kft., Mosonmagyaróvár, Hungary), and the absorbance was measured at 276 nm. Dissolution experiments were done with three replicates [80].

To compare the dissolution data of the different samples, similarity or difference factors were calculated; as a model-independent approach, the difference, *f*1 factor and similarity, and *f*2 factor was calculated for each sample:

$$f1=\frac{\sum_{j=1}^{n}\left(Rj-Tj\right)}{\sum_{j=1}^{n}Rj}\times \;100$$

where *n* is the number of samples and *Rj* and *Tj* are the percent dissolved of the reference and the test products at time point j.

$$f2=50\;\times \;\log\left\{{\left(1+\left(\frac{1}{n}\right)\sum_{j=1}^{n}wj\;{\left(Rj-Tj\right)}^{2}\right)}^{-0.5}\;\times \;100\right\}$$

where *wj* is an optional weight factor.

For the determination of release kinetics, the dissolved API amount was fitted to zero-order and first-order model equations:

$$Q=Q_{0}+k_{0}t$$

$$Q_{t}=Q_{0}e^{-k_{1}t},$$

where *Q* is the amount of drug released at time *t*, *Q*_0_ is the initial amount of the drug, and *Q_t_* is the amount of drug remaining at time *t*. *k*_0_ and *k*_1_ are the kinetic constants for the zero-order and first-order models, respectively [77].

#### 4.2.6. Cytotoxicity Experiments

##### Sterilization

The 3D-printed samples were immersed in 70% (*v/v*) ethanol in a laminar air flow (LAF) cabinet for 12 h, and they were individually placed to sterile medical-grade paper for drying [81].

##### Cell Culture

The colon adenocarcinoma (Caco-2) cell line was received from the European Collection of Cell Cultures (ECACC, Salisbury, UK, catalogue no. 86010202). Caco-2 is a well-established cell culture from ECACC and the protocol is based on the authors’ research [82]. Cells were seeded in plastic cell culture flasks (Thermo Fisher Scientific Inc., Budapest, Hungary) in DMEM medium supplemented with 3.7 g/L NaHCO3, 10% (*v/v*) heat-inactivated fetal bovine serum (FBS), 1% (*v/v*) nonessential amino acid solution, 1% (*v/v*) l-glutamine, 100 IU/mL penicillin, and 100 µg/mL streptomycin at 37 °C in an atmosphere of 5% CO_2_. For the cytotoxicity experiments, cells with 20 to 40 passages were used. The culture medium was replaced with fresh every three or four days [83].

##### MTT Cell Viability Assay

Cells were seeded on flat-bottomed 96-well tissue culture plates at a density of 10^4^ or 3 × 10^4^ cells/well. After separate sterilization of the test samples, they were put in sterile centrifuge tubes and immersed in 2 mL of DMEM medium. The samples were stored in a cell incubator at 37 °C. The test was performed on days 4, 8, and 12, and the samples were stored under the same conditions. The first step of the MTT assay was to remove the culture media from the cells, and then the cells were treated with 200 µL of the test sample solution and finally incubated for 30 min. After the incubation, the samples were removed, and the cells were washed with 200 µL PBS solution/well. Then, the cells were incubated with 100 µL 0.5 mg/mL MTT dye (3-(4,5-dimethylthiazol-2-yl)-2,5-diphenyl-2*H*-tetrazolium bromide dye) for at least 3 h. Finally, the formazan crystals were dissolved in acidic isopropanol (isopropanol: 1.0 N hydrochloric acid = 25:1). The absorbance was measured at 570 nm against a 690 nm reference with a FLUOstar OPTIMA Microplate Reader (BMG LABTECH, Offenburg, Germany). Cell viability was expressed as the percentage of the untreated control [40].

#### 4.2.7. Statistical Analysis

Data were analyzed using GraphPad Prism (version 7.0; GraphPad Software, Inc., San Diego, CA, USA) and presented as means ± standard deviation (SD). The dissolution results were examined with an unpaired *t*-test to compare the dissolved API amount (%) at 2 and 24 h. Design-Expert^®^ software version 10.0 was used for the historical statistical data analysis of the dissolution results. If it is not mentioned in the method description, then the experiments were carried out in triplicate [40].

## 5. Conclusions

Our implantable drug delivery system, prepared by FDM 3D printing, does not require hot-melt extrusion and can be directly printed with all kinds of API. Throughout the preparation, neither the high printing temperature (215–270 °C) nor the ventilation had an impact on the API stability, as confirmed by TG/DSC investigations.

FDM technology can be used for the manufacturing of 3D-printed implantable drug delivery systems with the required dissolution profile. The design of the sample and the printing parameters have an important role to play in optimizing the dissolution profile. The API is dissolved through the pores in the upper and lower surfaces of the samples, as confirmed by the SEM and microCT images. With the increase in size, the surface area is higher and the dissolution is faster due to the higher number of pores. The dissolution profile can be modified by a change of infill percentage.

For implantable systems, cytotoxicity measurements are compulsory. All samples are considered nontoxic based on the ISO standard, and these results are a good prediction of the in vivo data. Therefore, these samples can be selected for further in vivo and/or human studies.

PLA, antibacterial PLA, PETG, and PMMA polymers are all applicable for the manufacture of drug carriers, but PLA and antibacterial PLA are suggested as the most appropriate carrier systems. Our developed printing technique is easily usable during surgical interventions. Personalized medications can be immediately manufactured in the operating room during the surgery and the most appropriate combination of APIs (for example, anti-inflammatories or antibiotics) can be prepared at the patient’s bedside.

## Figures and Tables

**Table 1:** The samples’ average weight (mg) with standard deviation (SD) and the average content uniformity (mg) results with SD. Polylactic acid (PLA), antibacterial PLA (Anti), polyethylene terephthalate glycol (PETG), and poly-(methyl methacrylate) (PMMA) were measured with different diameters (16, 19, and 22 mm) and infill percentages (0%, 5%, 10%, or 15%). The nomenclature of the samples was as follows: first, the polymer used (e.g., PLA), then the diameter of the sample (e.g., 16), and finally the infill percentage (e.g., 0).

Sample	Weight		Content Uniformity	
	Average (mg)	±SD	Average (mg)	±SD
PLA_16_0	346.93	5.39	29.45	0.89
PLA_19_0	476.44	2.74	29.87	0.75
PLA_22_0	608.79	1.45	29.60	0.83
PLA_22_5	635.87	1.65	29.94	0.47
PLA_22_10	646.04	4.81	29.75	0.73
PLA_22_15	690.85	5.34	29.86	0.86
Anti_16_0	344.08	4.00	29.53	0.92
Anti_19_0	468.19	3.48	29.78	1.0
Anti_22_0	617.77	3.74	29.56	0.82
PETG_16_0	354.17	2.40	29.72	0.45
PETG_19_0	513.55	0.54	29.99	0.67
PETG_22_0	648.54	1.86	29.68	0.81
PMMA_16_0	366.49	3.61	29.66	0.36
PMMA_19_0	527.96	2.57	29.53	0.63
PMMA_22_0	615.87	2.84	29.91	0.78

**Table 2:** Degradation test results for the polylactic acid (PLA) and antibacterial PLA (Anti) samples with different diameters (16, 19, and 22 mm) and infill percentages (0%, 5%, 10%, or 15%). The average of the measured weight (mg) with standard deviation (±SD) can be seen right after printing and in the 1st, 2nd, 4th, 6th, and 8th weeks. During the examination period, our samples did not degrade.

Sample	Measured Weight (mg) ± SD					
	After Printing	1st Week	2nd Week	4th Week	6th Week	8th Week
PLA_16_0	315.44 ± 0.57	315.32 ± 0.42	314.82 ± 0.22	314.59 ± 0.84	315.93 ± 1.34	314.75 ± 0.56
PLA_19_0	446.87 ± 0.98	446.47 ± 0.88	446.53 ± 1.18	446.43 ± 0.58	445.86 ± 0.18	445.42 ± 0.76
PLA_22_0	576.96 ± 1.45	577.05 ± 0.72	576.37 ± 0.48	577.04 ± 0.33	577.04 ± 0.33	576.53 ± 0.18
PLA_22_5	603.65 ± 0.12	602.72 ± 0.27	602.27 ± 0.34	603.74 ± 0.37	602.72 ± 1.05	603.52 ± 1.05
PLA_22_10	615.98 ± 2.41	615.49 ± 0.38	615.92 ± 1.58	615.63 ± 0.81	615.07 ± 0.23	615.07 ± 0.45
PLA_22_15	660.57 ± 1.34	659.59 ± 1.14	660.39 ± 0.56	659.79 ± 0.34	660.82 ± 1.54	660.03 ± 0.72
Anti_16_0	313.82 ± 1.21	312.28 ± 0.51	313.03 ± 0.93	313.75 ± 0.63	312.34 ± 0.25	312.06 ± 0.57
Anti_19_0	437.19 ± 0.97	436.97 ± 0.84	437.03 ± 0.14	436.13 ± 0.59	436.94 ± 0.37	437.42 ± 0.70
Anti_22_0	586.82 ± 0.75	586.42 ± 0.55	585.97 ± 1.25	585.36 ± 0.74	586.22 ± 1.16	586.34 ± 0.04

**Table 3:** Four polylactic acid (PLA) samples with 16 mm diameter and 0%, 5%, 10%, or 15% infill amounts were examined with Raman spectroscopy to determine the diclofenac sodium amount in the cavities. The sample with 0% infill had only one cavity. In the other samples, the diclofenac amount in the six cavities was determined. The total API amount and the amount within the cavities was expressed in milligrams (mg). The total weight of the diclofenac was around 30 mg. For PLA_16_5, PLA_16_10, and PLA_16_15, ~75% of the whole API amount was in cavities 1, 2, and 3.

Sample	Weight of the Measured Diclofenac (mg) in the Cavity						Total Weight (mg) of Diclofenac
	1st Cavity	2nd Cavity	3rd Cavity	4th Cavity	5th Cavity	6th Cavity	
PLA_16_0	28.04 ± 0.15	-	-	-	-	-	28.04 ± 0.15
PLA_16_5	4.73 ± 0.04	7.87 ± 0.18	11.32 ± 0.71	2.61 ± 0.31	0.19 ± 0.005	1.34 ± 0.09	28.06 ± 0.22
PLA_16_10	10.89 ± 0.22	5.58 ± 0.16	11.76 ± 0.29	2.32 ± 0.17	1.87 ± 0.04	0.29 ± 0.13	32.71 ± 0.17
PLA_16_15	12.12 ± 0.10	6.41 ± 0.05	6.08 ± 0.004	3.10 ± 0.14	1.85 ± 0.20	0.29 ± 0.31	29.85 ± 0.13

**Table 4:** Diffusion rate, flux, dissolved API amount (%), and *t*-test results of the PLA, antibacterial PLA, PETG, and PMMA samples. The diffusion rate was calculated as an average between 0‒2 and 2‒24 h from the sample concentration at the adequate sampling time. The diffusion rate results were deducted from the surface area of the samples (16, 19, or 22 mm) to get the flux results to characterize the dissolved API amount through the surface. The dissolved API amount (%) results at 2 h and 24 h can be also found in the table and the other sampling time results in Table A1 with the standard deviation results. The samples’ *t*-test results can also be found in the table. For this calculation, every sample’s dissolved API amount result at 2 h and 24 h were analyzed by a *t*-test. The F test was nonsignificant in all cases.

Sample	Diffusion Rate (µg·mL^−1^·h^−1^)		Flux (µg·cm^−2^·h^−1^)		Dissolved API Amount (%)		*t*-Test Result
	Average 0–2 h	Average 2–24 h	0–2 h	2–24 h	2 h	24 h	2 h vs. 24 h
PLA_16_0	2.57	1.08	0.49	0.21	16.64	90.12	****
PLA_19_0	13.00	0.21	1.83	0.03	80.04	93.33	*
PLA_22_0	21.70	0.25	2.33	0.03	82.95	97.31	**
PLA_22_5	10.15	0.25	1.09	0.03	55.66	70.85	****
PLA_22_10	4.26	0.33	0.46	0.04	31.61	51.26	***
PLA_22_15	10.07	0.36	1.08	0.04	66.73	87.53	***
Anti_16_0	7.42	0.85	1.42	0.16	48.61	97.48	****
Anti_19_0	19.63	0.18	2.76	0.02	60.71	71.34	***
Anti_22_0	31.15	0.19	3.35	0.02	86.47	95.73	***
PETG_16_0	9.43	0.06	1.80	0.01	55.65	59.96	ns
PETG_19_0	28.62	0.08	4.03	0.01	94.22	99.49	***
PETG_22_0	7.76	0.40	0.83	0.04	57.70	79.77	****
PMMA_16_0	17.56	0.26	3.36	0.05	81.38	96.39	****
PMMA_19_0	17.18	0.00	2.42	0.00	97.23	97.14	ns
PMMA_22_0	21.77	0.12	2.34	0.01	92.95	99.48	*

*, **, *** and **** indicate statistically significant differences at *p* < 0.05, *p* < 0.01, *p* < 0.001 and *p* < 0.0001, respectively.

**Table 5:** The kinetic analysis of the dissolved samples. Drug release data were fitted to zero-order and first-order kinetic models for 0‒24 h, 0‒2 h, and 0‒X h. Samples labeled with * represent results that cannot be fitted to 0‒24 h or 0‒2 h, so an individual time point was determined. In the case of Anti_19_0 and PETG_19_0, X = 0.5 h, and Anti_22_0, X = 0.75 h.

Sample	Zero-Order Kinetics	First-Order Kinetics	Zero-Order Kinetics	First-Order Kinetics	* Zero-Order Kinetics	* First-Order Kinetics
	0–24 h	0–24 h	0–2 h	0–2 h	0–X h	0–X h
PLA_16_0	0.98	0.85	0.98	0.97	-	-
PLA_19_0	0.56	0.71	0.98	0.99	-	-
PLA_22_0	0.52	0.72	0.89	0.98	-	-
PLA_22_5	0.60	0.68	0.97	0.99	-	-
PLA_22_10	0.68	0.71	0.93	0.94	-	-
PLA_22_15	0.61	0.73	0.98	0.99	-	-
Anti_16_0	0.73	0.93	0.97	0.94	-	-
* Anti_19_0	0.43	0.60	0.65	0.68	0.91	1.00
* Anti_22_0	0.36	0.65	0.64	0.80	0.85	0.98
PETG_16_0	0.51	0.49	0.99	0.97	-	-
* PETG_19_0	0.29	0.67	0.60	0.77	0.95	1.00
PETG_22_0	0.61	0.67	0.98	0.97	-	-
PMMA_16_0	0.54	0.80	0.88	0.90	-	-
PMMA_19_0	0.45	0.40	0.93	0.95	-	-
PMMA_22_0	0.47	0.45	0.90	0.98	-	-

**Table 6:** Properties of the commercially available filaments.

Properties	Method	PLA	Antibacterial PLA	PETG	PMMA
Specific gravity (g/cm^3^)	D792	1.24	1.24	1.29	1.17
Heat distortion temperature at 0.45 MPa (°C)	D790	55	80‒90	68	106
Glass Trans. temperature (°C)	D3418	55‒60	55‒60	80	105
Tensile strength (MPa)	ISO 527	60	66	53	90
Tensile elongation (%)	ISO 527	6.00	3.31	4.01	15.0
Tensile modulus (MPa)	ISO 527	3800	4400	2040	2900
Notched Izod impact (kJ/m^2^)	ISO 180	16	118	4.5	6.4

**Table 7:** Properties of the 3D-printed filaments.

Properties	Method	PLA	Antibacterial PLA	PETG	PMMA
Tensile strength (MPa)	ISO 527	31.6	33.0	43.0	83.0
Tensile modulus (MPa)	ISO 527	1800	2300	2800	3200
Notched Izod impact (kJ/m^2^)	ISO 180	2.6	3.8	9.4	2.0

**Table 8:** Printing characteristics of the samples.

Filament Type	PLA	Antibacterial PLA	PETG	PMMA
Filament Diameter (mm)	1.75	1.75	1.75	1.75
Extruder Nozzle Diameter (µm)	400	400	400	400
Infill Percentage (%)	0, 5, 10, 15	0, 5, 10, 15	0, 5, 10, 15	0, 5, 10, 15
Extrusion Temperature (℃)	215	215	250	270
Bed Temperature (℃)	60	60	90	110
Layer Thickness (µm)	200	200	200	200

## Appendix A

**Table A1:** The dissolved API amount (%) at every sampling time (h) and the standard deviation (±SD) results can be found in Table A1.

Sample	PLA_16_0		PLA_19_0		PLA_22_0		PLA_22_5		PLA_22_10		PLA_22_15		Anti_16_0		Anti_19_0		Anti_22_0		PETG_16_0		PETG_19_0		PETG_22_0		PMMA_16_0		PMMA_19_0		PMMA_22_0	
Sampling time (h)	Dissolved API (%)	±SD	Dissolved API (%)	±SD	Dissolved API (%)	±SD	Dissolved API (%)	±SD	Dissolved API (%)	±SD	Dissolved API (%)	±SD	Dissolved API (%)	±SD	Dissolved API (%)	±SD	Dissolved API (%)	±SD	Dissolved API (%)	±SD	Dissolved API (%)	±SD	Dissolved API (%)	±SD	Dissolved API (%)	±SD	Dissolved API (%)	±SD	Dissolved API (%)	±SD
0	0	0	0	0	0	0	0	0	0	0	0	0	0	0	0	0	0	0	0	0	0	0	0	0	0	0	0	0	0	0
0.083	0.55	0.34	3.46	1.24	20.23	6.07	3.59	1.02	0.13	0.12	0.59	0.13	5.7	3.37	17.97	2.27	36.5	4.78	5.79	3.19	20.91	4.97	1.72	0.5	7.67	1.17	3.54	0.71	14.1	1.84
0.25	2.59	0.4	11.55	0.89	24.45	3.81	11.97	1.3	0.46	0.3	6.94	1.54	8.02	4.23	30.03	1.82	46.67	3.21	8.51	1.31	53.48	2.79	3.57	1.11	14.54	1.31	9.86	1.03	26.57	1.47
0.5	2.55	0.34	19.68	3.95	35.44	3.07	17.12	1.22	1.72	0.71	14.74	2.98	13.01	2.94	42.96	1.34	62.22	3.26	12.83	2.06	76.07	1.86	7.69	0.84	22.51	2	18.54	3.25	32.76	2.02
0.75	5.4	0.46	35.66	10.96	49.04	3.14	26.21	2.26	6.31	1.47	29.32	2.37	16.39	2.54	56.85	1.6	77.44	3.42	18.58	4.42	90.47	1.35	18.17	1.62	37.04	12.42	38.28	6.32	57.01	3.08
1	9.6	0.88	49.66	3.37	67.22	1.73	37.27	1.66	18.75	2.75	42.43	1.4	18.91	1.26	60.96	1.73	84.59	3.1	30.12	9.25	91.29	0.83	29.23	1.1	71.8	0.83	74.17	4.62	75.32	2.99
2	16.65	0.92	80.04	6.51	82.95	1.24	55.67	0.43	31.61	2.83	66.73	1.72	48.61	1.41	60.71	1.09	86.47	1.17	55.65	5.32	94.22	0.43	57.7	0.88	81.38	0.43	97.23	1.1	92.95	3.41
4	22.9	1.02	84.5	2.48	91.53	2.8	62.3	2.05	42.65	2.28	73.99	1.38	74.9	2.25	59.66	1.69	87.6	0.96	61.55	0.95	96.33	1.69	73.75	1.55	82.85	0.56	98.83	0.61	90.6	2.15
6	30.49	1.55	88.14	2.4	94.45	5.32	63.94	2.89	42.22	1.66	82.99	3.48	87.33	1.82	61.34	1.73	88.51	0.37	61.97	1.42	97.94	1.52	75.29	1.06	85.44	0.88	98.87	0.87	99.96	1.49
8	33.85	1.19	88.06	3.52	94.37	4.67	65.89	1.45	45.08	0.76	83.33	2.94	83.09	0.33	63.76	0.84	90.3	1.2	62.23	2.63	97.86	0.52	76.96	1.01	89.45	0.81	99.07	0.99	99.19	2.42
12	43.6	1.96	90.3	4.52	93.9	3.43	68.79	0.35	46.6	1.64	84.91	1.4	86.11	0.85	64.64	2.05	85.56	3.86	63.41	4.29	98.48	0.99	75.59	1.66	91.09	1.99	98.63	1.21	99.3	0.38
14	47.82	2.2	92.98	2.72	94.42	2.09	68.44	0.42	48.95	1.61	86.87	1.6	92.63	3.3	66.55	1.56	87.23	2.01	61.24	1.22	97.64	2.43	78.3	1.71	89.89	1.58	99.53	0.57	98.05	1.78
16	54.09	1.09	92.26	2.91	96.53	0.62	68.61	0.46	49.14	1.2	86.94	2.78	93.28	1.3	68.03	1.65	88.35	1.35	59.89	1.63	98.03	1.7	80.25	1.26	92.07	1.46	98.64	0.41	98.85	0.24
18	61.1	1.55	90.35	3.26	94.54	2.06	68.79	1.33	49.95	1.91	86.08	1.09	94.37	0.78	68.35	0.77	93.47	2.73	60.96	2.7	97.82	2.3	78.16	1.25	93.36	1.17	98.68	1.66	97.67	0.5
20	69.49	3.05	91.29	1.82	95.15	1.76	70.17	1.59	48.95	0.94	86.74	2.94	95.09	1.67	69.98	1.43	93.34	1.74	59.4	1.18	98.49	1.16	77.59	2.78	93.8	1.22	96.59	3.74	99.28	0.55
24	90.12	2.42	93.33	0.7	97.31	2.72	70.85	1.46	51.26	1.5	87.53	1.56	97.48	1.7	71.34	1.57	95.73	0.89	59.96	2.81	99.49	0.57	79.77	1.36	96.39	1.14	97.14	1.37	99.49	0.38

**Table A2:** Pairwise comparison results of the dissolution profiles can be found in Table A2: the f1 value of the difference factor calculation and f2 value of the similarity value calculation.

Pairwise Comparison of Dissolution Profiles			
Sample 1 vs.	Sample 2	f1	f2 (%)
PLA_16_0	PLA_19_0	51.47	17.47
PLA_16_0	PLA_22_0	56.63	14.22
PLA_19_0	PLA_22_0	10.63	48.21
PLA_22_0	PLA_22_5	32.87	27.41
PLA_22_0	PLA_22_10	57.25	15.32
PLA_22_0	PLA_22_15	18.68	38.61
PLA_22_5	PLA_22_10	36.31	33.58
PLA_22_5	PLA_22_15	23.87	40.79
PLA_22_10	PLA_22_15	90.20	22.55
Anti_16_0	Anti_19_0	42.81	26.82
Anti_16_0	Anti_22_0	26.55	22.85
Anti_19_0	Anti_22_0	28.31	29.51
PETG_16_0	PETG_19_0	47.87	15.46
PETG_16_0	PETG_22_0	19.98	42.03
PETG_19_0	PETG_22_0	60.80	18.49
PMMA_16_0	PMMA_19_0	9.21	51.12
PMMA_16_0	PMMA_22_0	11.16	47.93
PMMA_19_0	PMMA_22_0	7.07	51.33
PLA_16_0	Anti_16_0	46.36	21.26
PLA_16_0	PETG_16_0	39.89	30.54
PLA_16_0	PMMA_16_0	53.23	16.39
Anti_16_0	PETG_16_0	40.30	29.59
Anti_16_0	PMMA_16_0	14.56	35.43
PETG_16_0	PMMA_16_0	34.99	26.13
PLA_19_0	Anti_19_0	37.73	30.33
PLA_19_0	PETG_19_0	22.72	26.15
PLA_19_0	PMMA_19_0	10.82	46.70
Anti_19_0	PETG_19_0	34.04	23.19
Anti_19_0	PMMA_19_0	37.22	24.35
PETG_19_0	PMMA_19_0	18.02	28.65
PLA_22_0	Anti_22_0	13.01	40.53
PLA_22_0	PETG_22_0	39.05	30.36
PLA_22_0	PMMA_22_0	5.85	61.29
Anti_22_0	PETG_22_0	47.96	22.59
Anti_22_0	PMMA_22_0	15.28	39.93
PETG_22_0	PMMA_22_0	31.10	26.85
